# Feasibility and Effectiveness of a Wearable Technology-Based Physical Activity Intervention in Preschoolers: A Pilot Study

**DOI:** 10.3390/ijerph15091821

**Published:** 2018-08-23

**Authors:** Wonwoo Byun, Erica Y. Lau, Timothy A. Brusseau

**Affiliations:** 1 Department of Health, Kinesiology and Recreation, University of Utah, Salt Lake City, UT 84112, USA; tim.brusseau@utah.edu; 2 School of Kinesiology, University of British Columbia, Vancouver, BC V6T 1Z4, Canada; erica.lau@ubc.ca

**Keywords:** physical activity, sedentary behavior, intervention, social ecological model, preschool

## Abstract

The purpose of this pilot study was to evaluate the feasibility and the effectiveness of an intervention that employed a technology-based physical activity (PA) monitoring system and teacher-regulated strategies to promote PA in preschoolers. A total of 93 preschoolers (53% girls, 4.7 years) from 5 child care centers were recruited for a one-week intervention and randomly assigned into control (2 centers, n = 45) or intervention (3 centers, n = 48) group. Key intervention components included: (1) wearable device-based, real-time monitoring of children’s PA by classroom teachers and (2) teacher-regulated strategies for providing more opportunities for PA. Sedentary behavior (SED) and PA were measured using accelerometers. Overall, children in the intervention group showed significantly lower level of SED (31.6 vs. 33.6 min/h) and higher level of total PA (28.4 vs. 26.4 min/h) than children in the control group, after adjusting for age, sex, race, parent education level, parent perception of their child’s PA, BMI, and childcare centers. Teachers in the intervention group reported that the intervention was highly feasible to be implemented in their current classroom settings. In conclusion, we observed high acceptability and initial effectiveness of the current intervention. Subsequent research at larger-scale is warranted to fully evaluate the effectiveness of the intervention strategies tested in this study.

## 1. Introduction

Childhood obesity rates have more than doubled during the past few decades with approximately one in every five American children and youth currently obese [1]. Among numerous factors contributing to the childhood obesity epidemic, low levels of physical activity (PA) has been found to be the only behavioral risk factor that is causally and consistently associated with excessive weight gains in children as young as preschool age (3–5 years) [2,3,4].

The current enrollment of 3- to 5-year-old children in preprimary programs (i.e., preschools or childcare centers) is approximately 12 million in the U.S. and the majority are enrolled in full-day programs spending up to 10 hours per typical weekday in childcare centers [5]. Unfortunately, although Institute of Medicine (IOM) specifically recommends at least 15 min/h of PA while children attending childcare centers [6], a large proportion of children enrolled in these programs do not engage in sufficient PA while attending center-based childcare settings [7,8]. Given the fact that the child’s early years are a critical period for developing positive health behaviors that track into adulthood, the center-based childcare setting should be considered a prime environment in which to promote PA. 

Interventions aiming to increase preschoolers’ PA have reported inconsistent findings [9,10,11,12] and have had great variations in the approaches across interventions [13,14,15]. A few recent interventions that have focused on the availability of PA opportunities during the preschool day (e.g., teacher-led exercise breaks) appear to have increased children’s PA [15,16,17]. It has been reported that successful implementations of PA interventions are significantly influenced by the contextual factors of childcare settings and could look very different across classrooms [18]. The common aspects of successful interventions are that they were completed with optimal levels of program implementation and contextual congruency [19]. This suggests that interventions should allow teachers the flexibility and the adaptability to determine when and how to provide PA opportunities that fit their particular classroom.

Providing teachers with instant feedback on children’s PA levels during the preschool day may be a feasible approach to help them accurately determine the necessity and timing in providing PA opportunities. Recent advances in sensor technology have facilitated the employment of wearable devices as promising tools to provide instant feedback on individual’s PA. A few recent interventions utilizing a combination of wearable devices and self-regulation strategies, such as self-monitoring and goal setting with feedback have been shown to be effective in increasing PA in both adults and adolescents [20,21,22]. To the best of our knowledge, however, the acceptability and the potential effects of such intervention strategies have not been tested in young children. Therefore, the purpose of this pilot study was to evaluate the feasibility and the effectiveness of an intervention that employed a wearable device-based, real-time PA monitoring system and teacher-regulated strategies to promote physical activity in preschool-aged children attending childcare centers. We hypothesized that an implementation of the intervention strategies proposed in the current study would be feasible and children’s PA levels would be greater in the childcare centers that implement the intervention when compared with those that do not.

## 2. Materials and Methods

### 2.1. Participants and Setting

**Figure 1:**
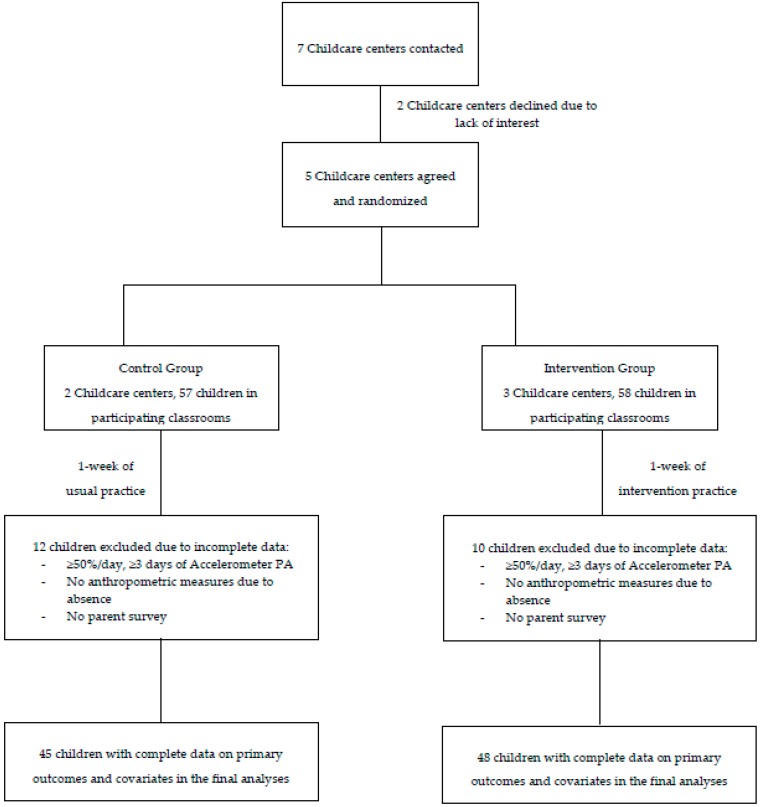
Flow diagram of participant recruitment and data analysis process.

The current pilot study employed a quasi-experimental, posttest-only design with two parallel groups. Childcare centers were invited to participate in this study if (1) the center was licensed by the National Association for the Education of Young Children (NAEYC), (2) the curriculum complied with the NAEYC standards, (3) teachers had early child education degrees and (4) teachers had direct contacts to children in the classroom all the time. In total, 5 childcare centers that met inclusion criteria were recruited and randomly assigned into either a control (n = 2) or an intervention group (n = 3). The number of participants per preschool-age classroom ranged from 16 to 20 in the control group and from 16 to 21 in the intervention group. Each preschool-age classroom was overseen by at least two teachers, thus the teacher-child ratio ranged from 1:8 to 1:10 in both the control and the intervention groups. After deletions of data with missing values, a total of 93 children (control: n = 45, intervention: n = 48) were included in the analysis (Figure 1). All data collection and measurements occurred during the same week at paired childcare centers (at least one control and one intervention center) in order to control for weather effects, which was considered as a potential confounder due to the timing of the year (March–April) and the geographical location of the study sites. Children who were physically disabled or unable to engage in regular PA as recommended by their pediatricians were not invited to this study. Written informed consent was obtained from children’s parents or guardians prior to participation in this study and the study was approved by the University’s Institutional Review Board (#HE16188). 

### 2.2. Intervention Overview

The intervention was developed based on the social ecological model (SEM) and health belief model (HBM). The SEM posits that individuals’ behaviors are influenced by factors operating at individual, social, organizational and policy levels [23]. In this case, children’s PA behaviors while attending the childcare centers are affected by their social environments, specifically teachers’ PA-related practices. Based on previous studies, providing PA opportunities was found to be an effective strategy in creating a supportive social environment to promote PA at childcare centers. The HBM theorizes that people’s beliefs about whether they are at risk for diseases or health problems as well as their perceptions of the benefits of and barriers to taking action and cues for action (e.g., reminders from others, media campaign) that will influence their likelihood to take a health-related action [24]. Previous research suggests that childcare teachers have inaccurate beliefs that children are sufficiently active, so they have low perceived needs to offer more opportunities for PA to children in their classroom [25]. Although some teachers may be aware of the health benefits of regular PA and the importance of promoting PA in young children, the lack of time and resources, low levels of self-efficacy and increased workload are common barriers that impede teachers from doing so [26,27].

The current intervention included a 1-week real-time PA monitoring system to provide teachers with instant feedback on children’s PA levels. Based on the feedback, teachers were asked to self-regulate their classroom strategies to provide more PA opportunities as needed. Teachers were given the autonomy to design the intervention strategies with expectations for encouraging children to be more physically active. The key principle was directing children to active opportunities that fit into usual classroom routines, such as offering outdoor playtime, dancing breaks and assisting teachers to clean up. These intervention components only require minimal teacher training, limited amounts of time for planning and implementation and little modification in classroom routine, thus teachers can flexibly adapt them. Moreover, the simple iterative feedback and self-regulating process will help teachers to develop accurate beliefs of children’s PA levels at childcare centers and increase knowledge and self-efficacy in determining when and how to regulate their classroom strategies to promote PA in response to their specific contexts. Ultimately, this approach will help to increase children’s PA levels during their stay at the childcare centers.

Fitbit Flex was chosen as the tool for real-time PA monitoring in this study. The usability and validity of Fitbit Flex have been established in preschoolers [28,29]. Each child’s PA data recorded by Fitbit Flex tracker was wirelessly transferred to the Fitbit’s application program interface (API) called Fitabase (Small Steps Lab, San Diego, CA, USA) that enables the user to monitor data from multiple Fitbit trackers in real time (Figure S1). Using a tablet PC, classroom teachers were able to monitor and receive instant feedback on each child’s time spent in sedentary behavior (SED) and PA throughout the day (i.e., total minutes of moderate-to-vigorous PA (MVPA)). This can ensure teachers have an accurate perception of children’s PA levels so that they can accurately determine the necessity of providing more PA opportunities. The feedback also acts as a cue for action reminding teachers to provide PA opportunities, particularly for children who have been inactive. Moreover, the feedback can help teachers to better understand children’s PA patterns across different time-periods so that they can identify which individual child and what time-period that requires the most changes. It can also provide an objective way for teachers to self-assess the effect of the strategies on children’s PA that they implemented in their classroom with the adaptability and compatibility. 

### 2.3. Procedures

The logic model of the current intervention is illustrated in Figure 2, which illustrated the logical relationship between resources, activities, outputs and outcomes. To put the intervention in place and support program delivery, researchers developed a partnership with the participating childcare centers. We also provided a training session (≈1 h) for teachers in the intervention group where research staff explained the primary purposes and implementation procedures of the current intervention to the teachers and answer any questions. Teachers were also provided with written instructions for assisting children to wear monitors, using tablet PCs to monitor children’s PA, guiding approaches to self-regulate their classroom strategies to promote PA and informing importance and health benefits of meeting the PA recommendations for preschoolers. Throughout the intervention, research staff maintained regular communications with the intervention teachers to support the implementation. During the intervention period, at the intervention childcare centers, upon their arrival at the classroom in the morning, teachers and parents were instructed to let children wear the Fitbit Flex activity trackers, which were fitted using age-appropriate wristbands, on their non-dominant wrist. Classroom teachers were then asked to check children’s PA level, which was monitored by the Fitbit. This provides instant feedback to teachers throughout the day to identify children who remained inactive, thus those children were directed and offered more opportunities for PA (e.g., dance to music or play outdoor). These iterative feedback and self-regulating process will help teachers to develop accurate beliefs and increase knowledge and self-efficacy in providing more active opportunities to children in their classrooms, thus increased children’s exposure to a more PA-promoting classroom environment. At the end of each day, children removed the Fitbit tracker right before leaving the center and kept it in their individual cubby until the next day. Children and teachers in the control group did not receive Fitbits or any other intervention materials but were asked to maintain their usual classroom routines. Childcare centers in the control group were informed that they would be eligible for implementing the same intervention program after all data collection is completed in the intervention group. 

### 2.4. Measures

#### 2.4.1. Sedentary Behavior and Physical Activity

SED and PA were measured using ActiGraph GT3X+ accelerometers (ActiGraph Corp., Pensacola, FL, USA) for children in both control and intervention groups. Children in the intervention group wore the GT3X+ accelerometer along with the Fitbit during the intervention period. A 15-s epoch was used to capture the spontaneous activities of preschool-aged children. An accelerometer was attached to the child’s hip using an elastic belt and worn for 5 consecutive days (Monday–Friday) while children attended their childcare center. Parents received information about the monitor and instructions for helping their child wear the monitor upon arrival at childcare center and for taking off the monitor before leaving for home. If children misplaced the accelerometers, temporary monitors were provided and data were later linked for each child. Children’s arrival and departure times from the childcare center were obtained from parental sign in/out sheet and applied in calculating precise hours that children attended the childcare centers. Raw accelerometer data was summarized into times spent in SED, MVPA and total PA (TPA) using age-appropriate cutpoints [30]. Due to variations in actual hours that children attend childcare centers, cumulative times spent in SED, MVPA and TPA were averaged on an hourly basis (min/hr). Children who wore the accelerometer for at least 50% of the center operating hours (≈5 h/day) and 3 valid days of the monitoring period were included in the current analyses.

#### 2.4.2. Demographic and Anthropometric Characteristics

Children’s age, sex, race/ethnicity and parent education as a surrogate indicator of socioeconomic status were reported by a parent or guardian using a parent survey. Each child’s height and weight were measured, and body mass index (BMI) percentile was calculated based on the values reported in the CDC growth charts [31].

#### 2.4.3. Parent Perception of Child’s Physical Activity

Each child’s parents or guardians completed a self-reported questionnaire, using 5-point Likert scale, to report their perceptions about the child’s level of PA compared with other children, the child’s amount of PA, the child’s enjoyment of PA and the child’s athletic coordination compared with other children of the same age and gender that were considered as potential covariates in the analyses of the current study.

#### 2.4.4. Intervention Acceptability

At the end of the study, parents of children in the intervention group completed a survey to provide their feedback on their child’s satisfaction and difficulty in wearing Fitbit activity trackers as well as the adequacy of monitoring PA in the childcare center that their child was attending. Teachers in the intervention centers also completed a 9-item survey, using 5-point Likert scale, to report their feedback on the satisfaction, difficulty, confidence, feasibility and effectiveness of implementing the intervention in their classrooms. 

### 2.5. Statistical Analyses

Descriptive statistics (Mean, SD and percent) for the participants were calculated and independent samples *t*-tests and Chi-square tests were used to examine differences in participant’s characteristics including accelerometer wear time variables between control and intervention groups. Differences in time spent in SED, MVPA and TPA between the control and the intervention group were determined using the linear mixed models (PROC MIXED procedure in SAS) adjusting for age, sex, race, parent education, BMI, the child’s enjoyment of PA, the child’s athletic coordination and parent perception of child’s PA and including the childcare center as a random effect in the models. All data was analyzed using SAS version 9.4 (SAS Institute, Cary, NC, USA).

## 3. Results

All characteristics, except for parents’ education level, were similar between the control and the intervention group (Table 1). 

**Figure 3:**
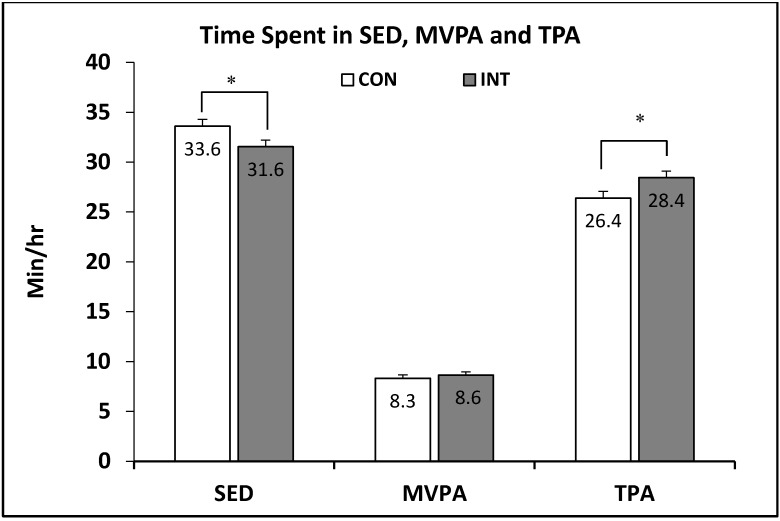
Average Time Spent in Sedentary Behavior Between Children Attending the CON and the INT Preschools. * Difference between the CON and the INT preschools adjusted for age, sex, race, parent education, parent perception of child’s PA, BMI and childcare centers (*p* < 0.05); Bars, standard error.

Overall, children in the intervention group showed significantly lower level of SED and higher level of TPA than children in the control group at post-intervention, after adjusting for age, sex, race, parental education level, parent perception of child’s PA, BMI and childcare centers (Figure 3). Although time spent in MVPA (Figure 3) and the percentage of hours that met the IOM recommendation criteria (≥15 min/h of TPA) tended to be higher among children in the intervention group (47.3%) than those in the control group (43.2%), the difference did not reach statistical significance. 

The results of teacher and parent surveys for the acceptability of the intervention are shown in Table 2. Both teachers and parents had very positive perceptions of the intervention, with the item mean scores ranging from 3.8 to 4.7 on a 5-point scale. On average, teachers in the intervention group checked children’s PA four times using the real-time monitoring system throughout the day and reported the intervention was effective and feasible for promoting PA in their classroom settings. Parents in the intervention group rated the intervention very adequate and effective and wearing Fitbit activity tracker very easy and enjoyable for their child. 

## 4. Discussion

The current study evaluated the feasibility and the effectiveness of an innovative intervention using a wearable technology, framed with the real-time monitoring of children’s PA and teacher-regulated strategies. Our findings showed that this intervention has received a high acceptability from teachers and parents and demonstrated its initial effectiveness. Considering significant amounts of time spent (up to 10 h/day) and low levels of PA at center-based early child education settings [5], our findings are of particular importance because they suggest that our innovative and easy-to-implement intervention strategies have considerable potential for increasing PA in preschool-aged children attending childcare centers.

In the current study, we observed that children in the intervention group spent 2.0 min/h less of SED and 2.0 min/h more of TPA compared to children in the control group (equivalent to standardized mean difference (SMD) of 0.7), which can be translated into 16 min/day or 90 min/week of reduction in SED and increase in TPA for children with an average attendance of 8 h/day at childcare centers. Given dose-response relationships between the levels of PA and health benefits in children, the observed intervention effects on SED and TPA can yield significant health benefits at long-term level. These findings are comparable to previous studies that reported the decreased SED ranged from 1.0 to 2.1 min/h (SMD range: 0.1–0.3) [32,33] and the increased TPA ranged from 0.4 to 1.4 min/h (SMD range: 0.1–0.5) among children in the intervention group [10,33,34]. Further, when we determined the effect of our intervention on meeting the PA recommendation while children attending childcare centers [6], the greater percentage of attendance hours in the intervention group was in compliance with the IOM recommendation. Given the fact that a large portion of preschoolers do not adhere to the recommended levels of PA and the paucity of consistent evidence on effective PA interventions in this age group, our results provide promise of success in developing evidence-based interventions aiming to promote PA in preschoolers. 

Despite the fact that the current intervention was not specifically designed to improve MVPA by prescribing particular activities at moderate-to-vigorous intensity but intended to promote overall PA via teachers’ self-regulation on children’s PA levels, we also observed the potential effect of our intervention on MVPA that, though statistically insignificant. Children in the intervention group engaged more MVPA by 0.6 min/h than the control group, which is similar to those reported in previous interventions (0.6–0.7 min/h) [10,35]. The comparable effects indicating that the intervention strategies used in this study have potential for increasing preschoolers’ MVPA. In support of this, the higher level of MVPA in the intervention group was not compensated by increased SED or decreased TPA in our sample. With consideration of a well-established dose-response relationship between MVPA and the risk of obesity in young children, [36,37,38] and the importance of establishing health-enhancing PA habits in early childhood, [39,40,41,42] we believe that the observed modest increase in MVPA would be meaningful practically to the long-term health of young children.

Previous PA interventions in childcare settings have relied on conventional approaches such as delivering an intervention program by research staff, prescribing structured PA curriculum, and/or modifying physical environments of preschools or childcare centers (e.g., alterations of playground and equipment), which are relatively more expensive, less flexible, and less scalable. In contrast, rather than require adherence to a rigid curriculum, our intervention strategies should allow teachers to be more flexible in integrating PA opportunities into daily lessons throughout the day, which may also improve the fidelity and the sustainability of the intervention because teachers prefer more adaptive and shorter bouts of lessons without major modifications of their current classroom environment [19,43,44].

Another significant merit of our intervention strategies, particularly the use of wearable technology, is the capability to remotely and objectively monitor primary outcomes (time spent in SED and PA) with reduced time and cost of data management as well as minimal risk of data loss, which ultimately reduces the overall cost of the intervention. Further, our technology-based intervention may appeal to the current technologically inclined generation of parents, children and teachers and yield greater scalability (e.g., online dissemination to distance locations) and fidelity (e.g., real-time monitoring of PA changes) by addressing inherent challenges found in conventional intervention programs.

Although the PA monitoring system (as a package of Fitbits, Fitabase API and tablet PC) utilized in this study was perceived as easy-to-use, adequate and feasible to be implemented by classroom teachers, a few modifications can be made to further enhance its utility in future trials. First, offer more choices of tablet PCs as some teachers expressed their preference on a type of tablet (e.g., iOS, Android, or Windows operating devices), though overall all teachers were comfortable using Microsoft Windows operating tablets. Second, customize the web interface of the Fitabase API based on teachers’ preference (i.e., Yes/No for meeting the IOM guidelines), despite the intuitiveness of the current user interface. For example, instead of using the child’s name, some teachers were willing to add the child’s picture in the API, so that they could tap it to open his/her activity data. Third, incorporate specific, yet adaptable, guidance on teacher-regulated strategies for promoting PA of children in their classroom. For example, the teachers participating in the intervention can be provided with daily lessons and materials involving active academic content to set up a “classroom activity center”, where children can be directed and ensured for active-play activities such as dance to action songs or Velcro darts with numbers and alphabets. Fourth, incorporate a family component into the current intervention. Considering the known promising effects of multi-level PA interventions, the current intervention has great potential for involving family members, thus it could enrich PA opportunities in the home setting as well. In fact, many parents of children that participated in our study expressed considerable interest in monitoring and regulating their child’s PA levels during non-childcare hours (e.g., before and after childcare hours, weekends). To those parents, we provided daily notes on their child’s PA levels as a courtesy and the receptiveness to being informed about their child’s PA levels throughout the day was excellent (e.g., as one parent put it, “Fun to find out how much my child moves throughout the day and then compare with his dad at suppertime”).

This study was conducted with several strengths and limitations worth noting. Significant strengths include an objective assessment of the primary outcome variables using the most widely validated accelerometer in preschool-aged children and an inclusion of carefully selected potential confounders in the statistical analyses. Some limitations to our pilot study design include a lack of pretest and a limited statistical power, thus an additional study with more rigorous research design is warranted to fully evaluate the effectiveness of the current intervention. A relatively short duration of the intervention period was another limitation of this study, so future research with a longer duration of intervention is necessary to evaluate the feasibility of long-term use of the Fitbit as an intervention tool in preschoolers. 

## 5. Conclusions

Our findings suggest that an intervention using real-time PA monitoring by classroom teachers and teacher-regulated strategies based on feedback from the PA monitoring was feasible and effective in promoting PA in preschool-aged children attending childcare centers. Considering the critical need to develop novel interventions for promoting PA to reverse the childhood obesity epidemic, subsequent research addressing valuable insights obtained from this pilot study is needed to confirm the observed initial effectiveness of the current intervention. 

## Figures and Tables

**Figure 2:** Logic model depicting the theory of change. Due to time and resources constraints, only indicators in bold-face were measured in the current study.

**Table:** 

Input	Activities	Output	Implementation outcomes	Program outcomes
Researchers provided preschool classroom teachers with the Fitbit Flex activity monitors together with the Fitabase monitoring software and intervention materials (i.e., instructions regarding the PA-monitoring system, self-regulation strategies to promote in-school PA and educational information on the importance and health benefits of meeting the current PA recommendations for preschool-aged children)	Researchers developed partnerships with preschool classroom-teachers provide training and technical assistance to help teachers to adopt the PA monitoring system into their classrooms	Teachers application of the PA-monitoring system into their classrooms: ○ Provided instructions for parents to help their children wear the monitor at childcare centers ○ **Used the system to monitor and receive feedback on children’s PA levels throughout the day** ○ Identified who’s active/inactive ○ Teacher-regulated instructional practices to provide more PA opportunities for children identified as inactive	Developed a more accurate perception of children’s in-school PA level Increased the number of in-school PA opportunities	Social-cognitive outcomes Increased perceived benefits of PA Decreased perceived barrier self-efficacy to provide PA opportunities at the centers **Behavioral outcomes** **Increased in-school PA levels**

**Table 1:** Descriptive Characteristics of Participants, Mean ± SD or Percent.

Characteristics	Group		
	CON	INT	*p*-Value
N	45	48	
Age (years)	4.8 ± 0.7	4.6 ± 0.7	0.06
Sex (%)			0.60
Boys	45.6	51.0	
Girls	54.4	49.0	
Race (%)			0.90
White	82.2	81.3	
Other	17.8	18.7	
Parent education (%) *			<0.05
<College	77.8	43.8	
≥College	22.2	56.8	
Parent perception of child’s PA			
Level of PA ^†^	3.4 ± 0.6	3.5 ± 0.8	0.85
Enough PA ^‡^	2.0 ± 0.6	1.9 ± 0.5	0.48
Enjoyment of PA ^#^	4.7 ± 0.4	4.6 ± 0.7	0.58
Athletic coordination ^$^	3.2 ± 0.7	3.5 ± 0.7	0.05
BMI (kg/m^2^)	16.3 ± 1.3	16.2 ± 1.3	0.85
BMI percentile	67.4 ± 26.9	65.6 ± 27.2	0.74
Overweight or obese (%)	33.3	29.2	0.67
Wear Time§			
Number of Days	4.7 ± 0.6	4.8 ± 0.5	0.48
Hours per Day	8.4 ± 1.0	8.2 ± 1.0	0.35

^†^ Child’s level of PA compared with others (1–5, 1: much less; 5 much more). ^‡^ Child’s amount of PA (1–4, 1: more than enough; 4: not enough). ^#^ Child’s enjoyment of PA (1–5, 1: not enjoyable; 5: very enjoyable). ^$^ Child’s athletic coordination (1–5, 1: much less; 5: much more). * Significantly different between the CON and the INT group (*p* < 0.05). ^§^ Number of days and number of hours that children wore accelerometers.

**Table 2:** Evaluation of Intervention Feasibility by Classroom Teachers and Parents, Mean ± SD or Percent.

Variables	Score Range (1–5)	Response Rate (%)	Response Score
Teacher evaluation (n = 8)			
How often did you check children’s PA throughout the day?	^†^ 1 = 0 times most days, 5 = 5 or more times most days	100	3.8 ± 0.7
In general, how did the children feel about the wearing activity monitors?	^‡^ 1 = hated it, 5 = loved it	100	4.3 ± 0.7
How easy or difficult was it to monitor children’s PA using the monitoring system throughout the day?	^#^ 1 = very difficult, 5 = very easy	100	4.1 ± 0.8
How easy or difficult was it to encourage children who had low PA to be more physically active?	1 = very difficult, 5 = very easy	88	3.9 ± 0.4
How easy or difficult was it to provide more opportunities for PA to children who had low PA?	1 = very difficult, 5 = very easy	88	4.6 ± 0.5
How would you rate the adequacy of the PA monitoring system from this study to be implemented in your classroom?	^$^ 1 = very inadequate/infeasible, 5 = very adequate/feasible	100	4.0 ± 0.3
How would you rate the feasibility of the monitoring system from this study to be implemented in your classroom?	1 = very inadequate/infeasible, 5 = very adequate/feasible	100	4.0 ± 0
To what extent did you feel confident to use the PA monitoring system in your classroom?	* 1 = very doubtful, 5 = highly confident	100	4.5 ± 0.5
How would you rate the effectiveness of using PA monitoring system to promote children’s PA in childcare settings?	^§^ 1 = very ineffective, 5 = very effective	100	4.4 ± 0.5
Teacher comments	- Wish there was a way to use the iPads in the room. - Wish there was an easier way to monitor the kids (i.e., click on child’s picture).		
Parent evaluation (n = 35)			
How did your child feel about wearing Fitbit activity monitors?	^‡^ 1 = hated it, 5 = loved it	73	4.3 ± 0.7
How easy or difficult was it to put and take off activity monitors on your child’s wrist?	^#^ 1 = very difficult, 5 = very easy	73	4.7 ± 0.6
How would you rate the adequacy of the PA monitoring system from this study to be implemented in the childcare center that your child is currently attending?	^$^ 1 = very inadequate/infeasible, 5 = very adequate/feasible	73	4.5 ± 0.6
How would you rate the effectiveness of the PA monitoring system from this study to promote your child’s PA in the childcare center that your child is currently attending?	^§^ 1 = very ineffective, 5 = very effective	73	4.4 ± 0.6
Additional comments	- Surprised at how quickly the kids embraced the program - Wish to know both a peer to peer context and an energy burned context. - Which to have a reference to compare my child’s data - Please provide us with information about the level of activity we should try for each day. - Hard to believe my child is mostly inactive. - So glad to participate as I wanted to buy an activity tracker for my child but the cost prevented from it. - Great learning experience for my child and enjoyed to talk about it with grandparents. - My child loved it. Amazing to see how much he really did move as I knew he had lots of energy.		

^†^ 1: 0 times most days; 2: 1 time most days; 3: 2 times most days; 4: 3 times most days; 5: 5 or more times most days. ^‡^ 1: hated it; 2: didn’t like it; 3: didn’t dislike or like it; 4: liked it; 5: loved it; ^#^ 1: very difficult; 2: somewhat difficult; 3: neither easy nor difficult; 4: somewhat easy; 5: very easy; ^$^ 1: very inadequate/infeasible; 2: somewhat inadequate/infeasible; 3: neither adequate/feasible nor inadequate/infeasible; 4: somewhat adequate/feasible; 5: highly adequate/feasible; * 1: very doubtful; 2: somewhat doubtful; 3: neither confident nor doubtful; 4: somewhat confident; 5: highly confident; ^§^ 1: very ineffective; 2: somewhat ineffective; 3: neither effective nor ineffective; 4: somewhat effective; 5: very effective.

## Supplementary Materials

The following are available online at http://www.mdpi.com/1660-4601/15/9/1821/s1, Figure S1: Fictional Sample Interface of Fitabase.
