# Supplementary figures and images for: Feasibility and Effectiveness of a Wearable Technology-Based Physical Activity Intervention in Preschoolers: A Pilot Study
Source: Int J Environ Res Public Health. 2018 Aug 23;15(9):1821. doi: 10.3390/ijerph15091821 (PMC6163401; doi:10.3390/ijerph15091821)

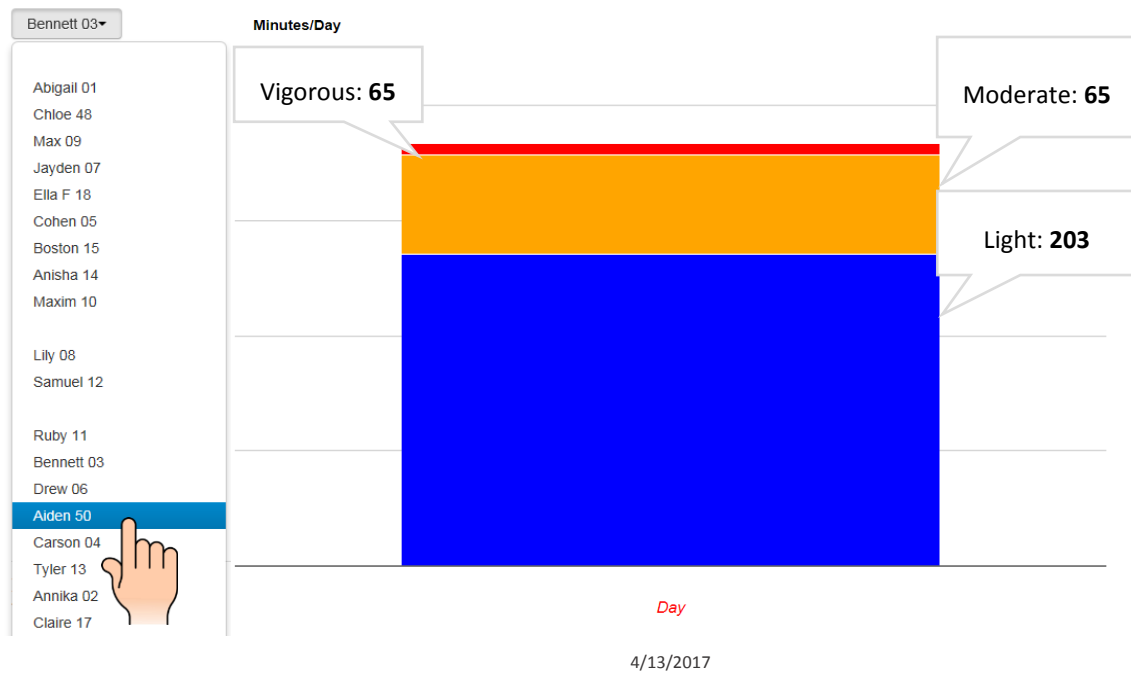

**Figure S1.** Fictional Sample Interface of Fitabase.

Supplement: Supplementary file 1 [file ijerph-15-01821-s001.pdf]
